# Analyzing the correlation between psychological capital in community nurses and their stress management and job satisfaction

**DOI:** 10.1186/s12912-025-03071-3

**Published:** 2025-05-02

**Authors:** Mostafa Shaban, Marwa Mamdouh Shaban, Huda Hamdy Mohammed, Majed Awad Alanazi, Hend Reda Ali Elkest

**Affiliations:** 1 Lecturer of Geriatric Nursing, Faculty of Nursing, Cairo University, Cairo, Egypt; 2 Lecturer of Community Health Nursing, Faculty of Nursing, Cairo University, Cairo, Egypt; 3 Faculty of Nursing, Cairo University, Cairo, Egypt; 4 Medical Surgical Nursing Department, College of Nursing, Jouf University, Sakaka, Saudi Arabia; 5 Community Health Nursing, Faculty of Nursing, Tanta University, Tanta, Egypt

**Keywords:** Psychological capital, Community nursing, Stress management, Job satisfaction, Nurse Well-being

## Abstract

**Background:**

Community nursing is marked by high stress due to direct patient interactions and varied work environments. Research highlights the significant role of psychological capital (PsyCap), which includes self-efficacy, hope, optimism, and resilience, in managing occupational stress and enhancing job satisfaction among health professionals.

**Objective:**

This study examines the associations between Psychological Capital, stress management, and job satisfaction among community nurses, exploring their collective impact on professional well-being.

**Methods:**

We employed a cross-sectional, correlational design with a convenience sampling method, recruiting 148 community nurses from Tanta University Educational Hospitals. The Compound Psychological Capital Scale (CPC-12), the Coping Orientation to Problems Experienced Inventory (Brief-COPE), and the Generic Job Satisfaction Scale (GJSS) were used for data collection. Pearson’s correlation and multiple regression analyses assessed the relationships and predictive values among the variables.

**Results:**

The study demonstrated significant positive correlations between PsyCap and job satisfaction (*r* = .44, *p* < .001) and between PsyCap and stress management (*r* = .39, *p* < .01). Multiple regression analysis showed that PsyCap accounted for 29% of the variance in job satisfaction and 26% in stress management effectiveness. Self-efficacy, hope, and optimism were significant predictors of job satisfaction, with self-efficacy showing the strongest association (β = 0.33, *p* = .007).

**Conclusion:**

Psychological Capital is associated with stress management and job satisfaction among community nurses, underscoring its importance in enhancing nurse coping mechanisms, reducing occupational stress, and improving job satisfaction. Practical implications include implementing training programs, resilience workshops, and mentorship initiatives to strengthen Psychological Capital among nurses, thereby improving workforce well-being and retention.

**Clinical trial number:**

Not applicable.

## Introduction

Community nursing occupies a pivotal role in healthcare systems worldwide, often acting as the first line of contact between patients and medical services in diverse settings [1]. From administering direct patient care and facilitating public health education, to coordinating with interdisciplinary teams, community nurses shoulder a wide range of responsibilities that are fundamental to societal well-being [2]. However, the complex nature of their roles exposes them to various stressors, which may include dealing with chronic illnesses in underserved populations, navigating high patient-to-nurse ratios, and contending with limited resources in rural or marginalized communities [3]. As such, managing stress effectively is critical not only for the personal health of these nurses but also for the quality of care they deliver. In recent years, researchers have begun to explore how psychological capital—an individual’s positive psychological state—can act as a buffer against occupational stress and enhance job satisfaction in health professionals [4, 5].

In healthcare settings, nurses are frequently exposed to emotional and physical demands that can lead to stress-related consequences, such as burnout, emotional exhaustion, and reduced job performance [6]. Community nurses, in particular, often face the added challenge of working in varying environmental conditions. Whether providing care in patients’ homes, local clinics, or community centers, they have to adapt to diverse socio-cultural contexts and resource constraints [7, 8]. These environments can exacerbate job-related strain, increasing the likelihood of stress accumulation and its potential effects on both mental and physical health [9]. Consequently, initiatives to improve nurses’ resilience and coping mechanisms have gained prominence as organizations seek to enhance workforce retention and improve healthcare outcomes [10]. Job satisfaction stands out among these efforts as a crucial factor influencing nurse turnover, patient satisfaction, and quality of care [11, 12]. Addressing the root causes of stress while bolstering positive psychological resources can significantly improve nurses’ commitment to their profession and their sense of well-being [13, 14].

The concept of psychological capital (PsyCap), popularized by Luthans et al., encompasses four core components: self-efficacy, optimism, hope, and resilience [15, 16]. Self-efficacy reflects an individual’s confidence in their ability to accomplish tasks and meet challenges successfully. Optimism entails a positive outlook on future outcomes, often leading to proactive coping strategies. Hope involves goal-directed energy and the planning to achieve those goals, while resilience is the capacity to bounce back from adversity [17, 18]. When nurses possess high levels of PsyCap, they are more likely to adopt adaptive coping strategies to handle work-related demands and stressors. This might manifest in more effective time management, better communication with colleagues and patients, and a positive, solution-focused mindset [19]. Moreover, these characteristics can be reinforced through targeted training, mentorship, and supportive organizational cultures [20, 21]. Therefore, investigating PsyCap among community nurses has significant implications for designing interventions that promote their mental well-being and job satisfaction [22].

Stress management in nursing practice can be approached through various theoretical frameworks, including the transactional model of stress, which posits that individual perceptions and appraisals of stressors affect the coping strategies deployed [23, 24]. Nurses high in PsyCap may view challenging situations as opportunities for professional growth rather than insurmountable obstacles [25]. They could, for instance, employ active problem-solving techniques, seek social support, or engage in self-reflective practices. Conversely, nurses with lower levels of PsyCap may be prone to maladaptive coping mechanisms, such as avoidance or rumination, which can intensify the experience of stress [26]. In the context of community nursing, where psychosocial demands are exceptionally pronounced due to patient diversity and unpredictable home-care environments, an understanding of how PsyCap influences stress management can illuminate pathways to foster greater occupational health [27, 28].

Job satisfaction is inherently tied to how nurses perceive their work environments, relationships with colleagues, and personal growth opportunities [29]. Positive psychological attributes can amplify these perceptions, enabling nurses to sustain commitment and enthusiasm even amidst daily challenges. Research has shown that nurses who experience higher job satisfaction are less likely to leave their positions, which is critical in addressing the ongoing global nursing shortage [30]. Moreover, satisfied nurses tend to demonstrate higher levels of patient-centered care, empathy, and engagement, ultimately improving patient outcomes. In community settings, these nurses often become pillars of public health, working closely with individuals over extended periods, thus reinforcing the importance of nurturing their psychological well-being [31].

Despite the growing body of evidence on the benefits of PsyCap in healthcare, research focusing specifically on community nurses is relatively sparse [32]. Much of the existing literature has traditionally emphasized hospital-based nursing roles, leaving gaps in understanding how positive psychological traits operate in community-based settings, where nurses might confront a broader spectrum of psychosocial and cultural challenges [1]. Given the evolving complexities of healthcare delivery—marked by shifts toward home-based care, increased chronic disease management, and the prioritization of preventive services—understanding these dynamics in community contexts is imperative [33]. By identifying the nature and extent of the correlation between psychological capital and stress management among community nurses, healthcare administrators and policymakers can introduce targeted programs designed to enhance mental resilience, reduce burnout, and increase job satisfaction [34, 35].

The aim of this study is, therefore, twofold: first, to analyze the correlation between psychological capital and stress management techniques employed by community nurses; and second, to determine how these factors jointly influence their overall job satisfaction. By examining these relationships, this research endeavors to contribute to a better theoretical understanding of how PsyCap operates in community healthcare contexts. Moreover, the findings are expected to offer practical insights for designing evidence-based interventions that reinforce the positive psychological resources of community nurses, ultimately helping them manage stress and remain satisfied and committed to their roles.

## Method

### Design

This study adopted a cross-sectional, correlational design to investigate the association between psychological capital in community nurses and their stress management strategies and job satisfaction. A cross-sectional approach was chosen to capture data at one point in time, allowing for a snapshot of the relationships among the variables of interest without attempting to establish causality. The correlational nature of the design enabled the researchers to quantify the strength and direction of the association between psychological capital, stress management, and job satisfaction. This design was considered appropriate for generating evidence that might inform future interventional studies targeting nurse well-being and retention.

### Setting

Data were collected at Tanta University Educational Hospitals, which are located in the Gharbia Governorate in Egypt. These hospitals provide comprehensive healthcare services to a broad population and include various units where community nurses are employed, such as outpatient clinics, home healthcare departments, and primary care centers affiliated with the university. This setting was deemed suitable because it provides a wide range of community nursing roles—covering preventive, curative, and rehabilitative care—and therefore offered a representative sample of nurses working in community-oriented contexts. By conducting the study in these diverse units, the researchers aimed to ensure that findings would be relevant to the multifaceted nature of community nursing practice.

### Sample and sampling

The sample size for this study was determined through a preliminary power analysis to ensure adequate statistical power for detecting meaningful relationships among the study variables. Using G*Power 3.1 software, the required sample size was calculated based on a medium effect size (*r* ≈ .3), a significance level (α = 0.05), and a power of 0.80, ensuring an 80% probability of detecting true effects while minimizing the risk of Type II errors. Given that Pearson’s correlation and multiple regression analyses were the primary statistical tests, a minimum of 120 participants was deemed necessary to detect moderate effect sizes with 95% confidence. To enhance statistical robustness and account for potential non-responses or missing data, the final sample size was increased to 148 community nurses. A convenience sampling method was employed, allowing for practical recruitment from Tanta University Educational Hospitals while capturing a diverse range of experiences among community nurses. Although convenience sampling may limit generalizability, ensuring an adequate sample size and utilizing validated measurement tools helped strengthen the reliability and applicability of the study findings. The inclusion criteria comprised the following: (1) community nurses who had been working at Tanta University Educational Hospitals for a minimum of six months, (2) those willing to participate and provide informed consent, and (3) those able to read and understand Arabic, as the instruments were administered in Arabic. Nurses on prolonged leave or those who declined to participate were excluded.

### Data collection tools

Three standardized and publicly available instruments with validated Arabic versions were used to measure the main variables, ensuring no copyright restrictions:

1. Compound psychological capital scale (CPC-12) The Compound PsyCap Scale-12 [36] is a brief, self-report scale that was developed for a set of two studies in Germany as a non-domain-specific measure of psychological capital, expanding the range of contexts usually covered by the standard domain-specific measure, the Psychological Capital Questionnaire [37]. Its primary purpose is to capture an individual’s overall positive psychological state, reflecting their ability to persevere toward goals, maintain a positive outlook, and rebound from adversity.
  The CPC-12 includes 12 items that are evenly distributed among the four dimensions of Psychological Capital:

1. **Self-efficacy**: Confidence in one’s abilities to handle work-related tasks and challenges.
2. **Hope**: Goal-oriented thinking and the perceived capability to devise pathways to achieve those goals.
3. **Optimism**: A positive attribution about succeeding now and in the future.
4. **Resilience**: The ability to recover quickly from setbacks or difficulties.

Each dimension is represented by three items, ensuring a balanced assessment of an individual’s Psychological Capital. The CPC-12 typically uses a 5-point Likert-type response format (1 = strongly disagree to 5 = strongly agree), After reverse-scoring any negatively worded items (if present in the version used), the total scale score is computed by summing all item responses. Higher scores indicate stronger overall Psychological Capital. Researchers may also calculate subscale scores for each of the four components if a more granular analysis is desired.

Initial validation studies reported good internal consistency, with Cronbach’s alpha estimates typically ranging from 0.80 to 0.90 across different populations. Convergent validity has been established through correlations with other measures of well-being, job satisfaction, and coping strategies. Construct validity was supported by factor analyses, which confirmed the four distinct but interrelated dimensions of PsyCap.

### Translation into Arabic Language

An Arabic version of the CPC-12 was developed through a forward-backward translation process by bilingual experts, ensuring semantic and conceptual equivalence. This process included independent forward translation into Arabic, backward translation into the original language, and subsequent review by a panel of experts to resolve any discrepancies. the Arabic CPC-12 has been tested over pilot of 15 nurses and demonstrated a Cronbach’s alpha of approximately 0.85 indicating strong internal consistency. Confirmatory factor analysis (CFA) has supported the four-factor structure, paralleling findings from the original validation. Moreover, significant positive correlations with measures of employee engagement, coping efficacy, and job satisfaction provided evidence for concurrent validity in Arabic-speaking populations.

2. The coping orientation to problems experienced inventory (Brief-COPE) (Arabic Version) The Brief COPE Scale was originally developed by Charles S. Carver in 1997 as an abbreviated version of the COPE Inventory [38]. Its primary aim is to evaluate a broad range of coping responses that individuals use when confronted with stressors. Unlike some other coping scales that focus on a single domain, the Brief COPE captures both adaptive and maladaptive strategies, making it highly applicable to healthcare settings where practitioners face multiple types of stress. The Brief COPE Scale was selected as it provides a comprehensive assessment of coping mechanisms rather than merely measuring stress levels. This distinction is crucial in understanding how community nurses manage occupational stress through various adaptive and maladaptive coping strategies. Given the diverse and often unpredictable challenges faced in community nursing settings, evaluating coping styles offers valuable insights into stress management effectiveness and resilience-building processes.

The Brief COPE Scale consists of 28 items, each describing a specific coping response. These items are categorized into 14 subscales, with two items per subscale. Some of the key coping strategies assessed by the scale include active coping, which involves taking direct action to address stressors; planning, which refers to strategizing ways to manage challenges effectively; and positive reframing, which entails reinterpreting stressful situations in a more optimistic light. Other subscales measure acceptance, which reflects acknowledging and coming to terms with difficult circumstances, and humor, which involves using humor as a coping mechanism. Additionally, the scale evaluates emotional support, which captures seeking comfort and reassurance from others, and instrumental support, which refers to seeking tangible assistance. Further, it assesses self-distraction, which involves diverting attention away from stressors, substance use, which examines reliance on drugs or alcohol as a coping method, and denial, which measures the tendency to avoid acknowledging stressors.

Because community nurses often employ diverse coping mechanisms—ranging from active problem-solving to emotional venting—this scale offers insight into which strategies are most prevalent and potentially effective in managing work-related stress.

Each item is rated on a 4-point Likert scale (1 = I usually don’t do this at all; 4 = I usually do this a lot). For each of the 14 subscales, the two relevant items are summed to yield a subscale score, with higher scores indicating a greater tendency to use that particular coping strategy. In research contexts, subscale scores can be analyzed individually, or similar subscales (e.g., those reflecting problem-focused vs. emotion-focused coping) may be aggregated as per the study’s objectives.

Carver’s original work and subsequent studies have consistently reported acceptable reliability coefficients for most subscales, generally above 0.60, with some subscales (e.g., Active Coping, Planning) showing Cronbach’s alpha values above 0.70. Test-retest reliability over short intervals has also been documented, supporting the scale’s stability.

The Brief COPE Scale has been translated into Arabic following a standard forward-backward translation methodology, ensuring cultural and linguistic appropriateness [39]. For non-commercial research purposes, the Arabic version is publicly available, allowing researchers in Arabic-speaking regions to evaluate coping strategies without incurring additional licensing costs.

3. Generic job satisfaction scale (GJSS; Arabic Version) The Generic Job Satisfaction Scale (GJSS) was developed by Macdonald and MacIntyre in 1997 to provide a succinct measure of overall job satisfaction that can be applied across different occupational groups [40]. The aim is to assess employees’ affective reactions to their job, tapping into global contentment rather than domain-specific dimensions such as salary satisfaction or supervisory support.

The GJSS is typically composed of 10 items (although some versions include minor item variations), each gauging an employee’s attitude toward their job. Sample items may address feelings of enthusiasm, boredom, pride, or fulfillment in daily work tasks. Because of its brief nature, the GJSS can be integrated easily into larger surveys without overburdening participants.

Items are usually scored on a 5-point Likert scale (1 = strongly disagree to 5 = strongly agree). Some items might be reverse-coded if negatively phrased. The total job satisfaction score is derived by summing all items, with higher scores reflecting greater overall satisfaction. Researchers can also compute an average scale score by dividing the sum by the total number of items. Macdonald and MacIntyre reported Cronbach’s alpha values in the range of 0.77 to 0.88 for the original English version, indicating high internal consistency. The scale has also shown good convergent validity, correlating positively with organizational commitment and negatively with turnover intentions in diverse work settings.

An Arabic translation of the GJSS is available for academic and clinical research purposes. As with the other tools, the translation process involved forward and backward translation steps, followed by expert panel reviews and pilot testing. The Arabic version retains the original scale’s structure, ensuring conceptual equivalence.

In studies applying the Arabic GJSS to various occupational groups, Cronbach’s alpha has frequently been reported above 0.80, suggesting robust internal consistency. Construct validity has been supported through significant associations with related job outcomes (e.g., turnover intention, perceived organizational support) and psychosocial constructs (e.g., work engagement). Because of its brevity and demonstrated psychometric soundness, the Arabic GJSS is well-suited for research exploring job satisfaction among healthcare professionals, including community nurses.

## Data collection procedure

Data were collected over a four-week period from **November 1, 2024 to November 30, 2024**. Prior to distributing the questionnaires, the researchers coordinated with hospital administration and nursing directors to schedule times that would minimally disrupt the nurses’ workflow. The study’s purpose, procedures, potential benefits, and voluntary nature were explained to prospective participants. Upon agreeing to partake, each nurse received a questionnaire package containing a cover page outlining instructions and assurance of confidentiality, followed by the three instruments in Arabic. Nurses were generally given 20–25 min during breaks or at the end of their shifts to complete the questionnaires. Completed surveys were returned in sealed envelopes to maintain anonymity and confidentiality. Reminder visits were made once a week for two weeks to enhance the response rate. By the end of the collection period, 148 fully completed questionnaires were obtained and deemed suitable for analysis.

### Data analysis

Once all questionnaires were collected, they were systematically inspected for completeness and any obvious discrepancies. Data were then coded and entered into the Statistical Package for the Social Sciences (SPSS) version 28.0. Prior to the main analysis, the dataset was cleaned by removing or addressing any missing values. Cases with excessive missing data were excluded listwise, while pairwise deletion was used for minor omissions to maximize data retention.

Descriptive statistics, including means, standard deviations, frequencies, and percentages, were computed to summarize the sample’s demographic profile and the distribution of main variables. Reliability of each tool was examined through Cronbach’s alpha, ensuring internal consistency. Normality assumptions were evaluated using the Kolmogorov–Smirnov test and histograms; no significant deviations were detected, indicating that Pearson’s correlation coefficient (r) would be appropriate for investigating the linear relationships among the three main variables.

Subsequently, correlational analyses were conducted to assess the strength and direction of associations between psychological capital, stress management strategies, and job satisfaction. A series of multiple regression models were then performed to determine how well psychological capital predicted both job satisfaction and the effectiveness of stress management, controlling for relevant demographic factors such as age, gender, years of experience, and work unit. Standard assumptions for multiple regression—linearity, independence of errors, homoscedasticity, and multicollinearity—were checked using scatterplots, the Durbin–Watson statistic, and variance inflation factors (VIF). A p-value of < 0.05 was used to denote statistical significance throughout all analyses.

### Ethical consideration

All study procedures were conducted in strict accordance with ethical guidelines for research involving human participants. Ethical approval was obtained from the Institutional Review Board (IRB) at the Faculty of Nursing, Tanta University (Approval Code: 524-9-2024) on October 7, 2024. Prior to data collection, information sheets were provided to potential participants, clearly detailing the study’s objectives, the voluntary nature of participation, and the option to withdraw at any point without adverse consequences. Written informed consent was obtained from each participant, ensuring they fully understood the study’s scope and any foreseeable risks or benefits.

Protecting participant privacy and confidentiality was paramount. Questionnaires were completed anonymously, and no identifying information (such as names or employee IDs) was collected. Paper-based surveys were stored in locked cabinets accessible only to the core research team, while electronic datasets were password-protected and backed up on secure storage devices. In accordance with the university’s data management policies, the research team will retain the raw data for five years following the study’s conclusion, after which time hard copies will be securely shredded and electronic files permanently deleted.

## Results

Participants were predominantly aged between 30 and 39 years (44.6%), with smaller but substantial proportions under 30 (33.1%) and 40 or above (22.3%). More than three-fifths were female (61.7%), and just over half reported being married (53.1%). Almost two-fifths of the nurses (34.4%) had between one and three years of experience, while 44.6% had four to seven years, and 20.9% reported eight or more years. Regarding educational level, nearly one-quarter held a Master’s degree (23.2%), reflecting a relatively well-qualified sample of community nurses (Table 1).

**Table 1:** Demographic characteristics of the study participants

Variable	Category	*n*	%
Age	< 30	49	33.1
	30–39	66	44.6
	≥ 40	33	22.3
Gender	Male	57	38.3
	Female	91	61.7
Marital status	Single	41	27.7
	Married	79	53.1
	Divorced	17	11.6
	Widowed	11	7.2
Years of experience	1–3	51	34.4
	4–7	66	44.6
	≥ 8	31	20.9
Educational level	Diploma	48	32.4
	Bachelor’s	66	44.6
	Master’s	34	23.2

Table 2 presents descriptive statistics for (a) the Compound Psychological Capital Scale (CPC-12) subscales and total score, (b) selected subscales of the Brief COPE Scale, along with an overall coping score, and (c) the Generic Job Satisfaction Scale (GJSS). The nurses demonstrated moderate to relatively high levels of Psychological Capital, with an average total CPC-12 score of 39.7 ± 6.1. Resilience and self-efficacy were slightly higher on average than hope and optimism. For stress management, the total Brief COPE mean was 53.3 ± 7.1, indicating moderate reliance on a range of coping strategies; subscales such as active coping and planning showed generally higher mean values than denial or other less adaptive strategies. Meanwhile, the mean job satisfaction score (33.7 ± 5.1) suggests that the participants were moderately satisfied with their work.

**Table 2:** Descriptive statistics of study variables

Variable	Mean (M)	SD	Possible range
**CPC-12 subscales**			
Self-efficacy	10.3	2.7	3–15
Hope	9.4	2.6	3–15
Optimism	9.8	2.8	3–15
Resilience	10.2	2.9	3–15
**Total CPC-12**	39.7	6.1	12–60
**Brief COPE subscales**			
Active coping	5.7	1.6	2–8
Positive reframing	4.9	1.4	2–8
Planning	5.8	1.7	2–8
Denial	2.9	1.2	2–8
**Total coping score**	53.3	7.1	28–112
**Generic job satisfaction**	33.7	5.1	10–50

Table 3 displays Pearson’s correlation coefficients (r) among the total CPC-12 score, total Brief COPE score, and the GJSS total score. Correlation coefficients (r) range from − 1.0 to + 1.0, with positive values indicating a direct relationship. All correlations were statistically significant at the 0.01 level or better. Psychological Capital (CPC-12) demonstrated a moderate positive correlation with both stress management (*r* = .39, *p* = .003) and job satisfaction (*r* = .44, *p* = .001). Similarly, higher coping scores were associated with higher job satisfaction (*r* = .36, *p* = .006). These findings suggest that nurses who exhibit greater Psychological Capital tend to employ more effective coping strategies and report higher levels of job satisfaction.

**Table 3:** Correlations among main study variables

Variable	1. CPC-12	2. Brief COPE	3. Job Satisfaction
1. CPC-12	—	0.39**	0.44***
2. Brief COPE		—	0.36**
3. Job Satisfaction			—

**p < .01; ***p < .001

A multiple linear regression analysis was conducted to determine how the four CPC-12 subscales (self-efficacy, hope, optimism, and resilience) predicted nurses’ job satisfaction, controlling for age, gender, and years of experience. Table 4 presents the unstandardized coefficients (B), standardized coefficients (Beta), t-values, and p-values for each predictor. The overall model fit (F) and adjusted R² are also shown. The regression model explained approximately 29% of the variance in job satisfaction (R² = 0.29), which is statistically significant (*p* < .001). Among the four dimensions of Psychological Capital, self-efficacy (*p* = .007), hope (*p* = .016), and optimism (*p* = .032) emerged as significant positive predictors of job satisfaction, while resilience (*p* = .092) had a weaker, non-significant influence in this model. These results indicate that believing in one’s capabilities, having a hopeful outlook, and maintaining optimism are key factors contributing to higher job satisfaction levels among community nurses.

**Table 4:** Multiple regression analysis predicting job satisfaction

Predictor	B	Beta	t	*p*
Self-efficacy	0.78	0.33	2.8	0.007
Hope	0.63	0.27	2.4	0.016
Optimism	0.48	0.21	2.2	0.032
Resilience	0.32	0.13	1.7	0.092
Model fit				

R² = 0.29; F(7, 140) = 7.2; *p* < .001

A second multiple regression was performed to examine how CPC-12 subscales predict overall stress management (total Brief COPE score), again controlling for age, gender, and years of experience. Table 5 summarizes the outcomes. This model accounted for 26% of the variance in nurses’ stress management strategies (R² = 0.26), with a significant overall fit (*p* < .001). Hope (*p* = .005) emerged as the strongest predictor, followed by self-efficacy (*p* = .011) and optimism (*p* = .025). Resilience did not reach statistical significance in predicting Brief COPE scores (*p* = .165). These findings reinforce the notion that specific positive psychological attributes—particularly hope and self-confidence—are closely tied to more adaptive coping behaviors in community nursing contexts.

**Table 5:** Multiple regression analysis predicting stress management (Brief COPE)

Predictor	B	Beta	t	*p*
Self-efficacy	0.54	0.29	2.6	0.011
Hope	0.66	0.34	2.9	0.005
Optimism	0.42	0.22	2.3	0.025
Resilience	0.18	0.09	1.4	0.165
**Model Fit**				

R² = 0.26; F(7, 140) = 6.9; *p* < .001

To explore whether total Psychological Capital differed by years of experience, a one-way ANOVA was performed using three groups: 1–3 years, 4–7 years, and ≥ 8 years of experience. Table 6 presents the group means, standard deviations, and the ANOVA test result. The ANOVA indicated a significant difference in CPC-12 scores across the three experience groups (F = 3.3, *p* = .041). Post-hoc analyses (not shown in the table) revealed that nurses with 8 or more years of experience had significantly higher Psychological Capital compared to those with fewer years, suggesting that prolonged engagement in community nursing may foster or be associated with a more robust positive psychological state.

**Table 6:** Total psychological capital by years

Years of experience	*n*	Mean (SD)	F	*p*
1–3	51	38.7 (5.9)	3.3	0.041
4–7	66	40.1 (6.2)		
≥ 8	31	41.3 (6.6)		

Table 7 presents key model fit indices and reliability statistics for the translated measurement tools, demonstrating strong psychometric properties. The χ²/df values for all three tools (CPC-12 = 2.87, Brief COPE = 2.42, GJSS = 2.51) fall within the acceptable range, indicating a reasonable model fit. The CFI and TLI values exceed 0.90, further confirming the good fit of the models, while the RMSEA values (CPC-12 = 0.062, Brief COPE = 0.058, GJSS = 0.056) remain below the 0.08 threshold, suggesting minimal model error. Additionally, the Cronbach’s alpha values for the total scales (ranging from 0.81 to 0.87) indicate strong internal consistency, with subscales maintaining acceptable reliability. While these findings support the validity and reliability of the Arabic-translated scales, future research could enhance the evaluation by providing subscale-specific CFA results, confidence intervals for RMSEA, or alternative fit indices such as SRMR or AGFI.

**Table 7:** Confirmatory factor analysis (CFA) fit indices and reliability statistics for the Arabic-translated measurement tools

Scale	χ²/df	CFI	TLI	RMSEA	Cronbach’s Alpha
CPC-12	2.87	0.93	0.91	0.062	0.85 (subscales: 0.78–0.83)
Brief COPE scale	2.42	0.92	0.90	0.058	0.81 (subscales: 0.68–0.79)
GJSS	2.51	0.94	0.92	0.056	0.87

**Fig. 1:**
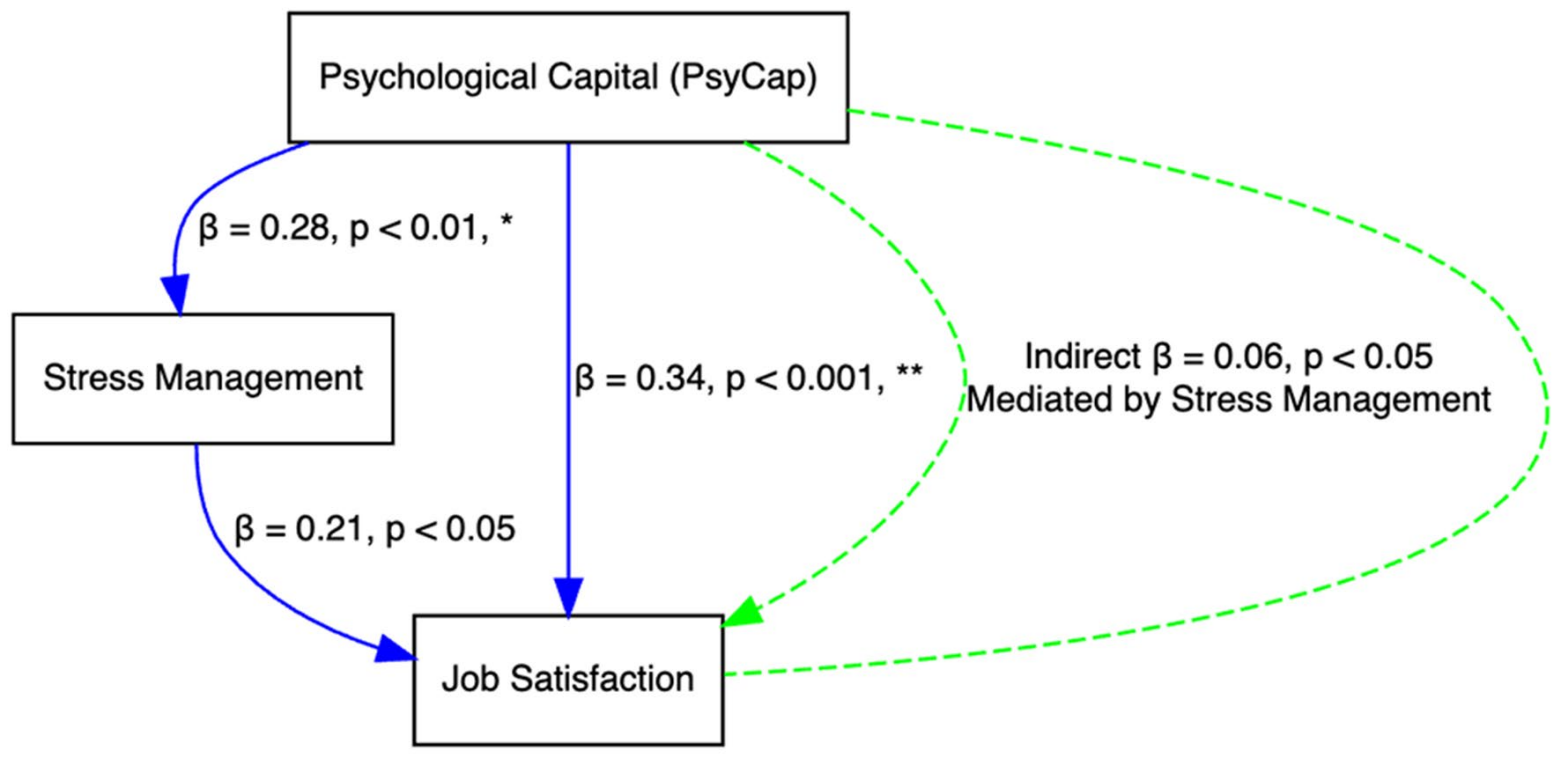
The path analysis model

The path analysis model results (Fig. 1) provide compelling evidence that Psychological Capital (PsyCap) significantly influences job satisfaction both directly and indirectly through stress management among community nurses. The strong direct relationship between PsyCap and job satisfaction (β = 0.34, *p* < .001) underscores the crucial role of intrinsic psychological resources like self-efficacy, resilience, hope, and optimism in fostering satisfaction at work. Similarly, PsyCap’s direct effect on stress management (β = 0.28, *p* < .01) suggests that higher levels of PsyCap enable nurses to effectively manage work-related stress, thereby enhancing their overall job satisfaction. The significant indirect effect of PsyCap on job satisfaction through stress management (β = 0.06, *p* < .05) further highlights the importance of coping strategies as mediators in this relationship. These findings suggest that interventions aimed at boosting psychological resources could substantially benefit job satisfaction by improving both direct psychological outcomes and stress management capabilities.

## Discussion

The results of this study shed light on the pivotal role that Psychological Capital (PsyCap) plays in influencing both stress management and job satisfaction among community nurses. Specifically, the data revealed a moderate-to-strong positive correlation between PsyCap and job satisfaction, as well as between PsyCap and stress management. These associations are particularly notable in the context of community nursing, which is characterized by high levels of occupational stress due to diverse work environments, limited resources in underserved areas, and the need to address the broad psychosocial needs of patients [19, 41]. The findings build upon earlier work suggesting that personal resources, such as self-efficacy and optimism, serve as buffers against occupational stress in healthcare settings [42–44].

One of the most significant observations from the multiple regression analyses was that, among the four components of PsyCap, self-efficacy, hope, and optimism emerged as strong predictors of job satisfaction and stress management [45, 46]. Although resilience did not reach significance in the final models, it still demonstrated a positive, albeit weaker, association. These results dovetail with prior research indicating that nurses with higher levels of self-efficacy often engage in more proactive coping strategies and exhibit lower turnover intentions [47]. High self-efficacy empowers individuals to view workplace stressors as challenges rather than threats, thereby mitigating emotional exhaustion and bolstering their commitment to patient care [48, 49]. Hope and optimism, both of which involve positive expectations about future outcomes, likely encourage solution-focused and adaptive coping mechanisms, consistent with the transactional model of stress [50]. Such a mindset can be critical for community nurses who frequently operate in less controlled, variable settings compared to their hospital-based counterparts [51].

The path analysis further underscored the importance of stress management as a mediating mechanism between PsyCap and job satisfaction. The direct effect of PsyCap on stress management suggests that individuals with stronger psychological resources are better equipped to cope with everyday occupational challenges, thereby preventing these demands from escalating into chronic stress. This notion is in harmony with the broader literature, which posits that positive psychological capacities can empower healthcare professionals to select active and adaptive coping strategies—such as seeking emotional or instrumental support—over more passive approaches like denial or disengagement [52, 53]. Importantly, the indirect effect of PsyCap on job satisfaction through stress management provides empirical evidence for the argument that job satisfaction in nursing does not solely hinge on external factors (e.g., salary, workload, or administrative support), but is also significantly shaped by internal, personal resources [4, 54, 55]. Thus, interventions aimed at strengthening PsyCap may foster an environment in which nurses are better able to manage stress, leading to higher levels of professional contentment and retention.

Another intriguing finding was that longer years of experience in community nursing were associated with slightly higher PsyCap scores. Nurses with eight or more years of experience reported more robust psychological resources than their less-experienced peers, suggesting that resilience, hope, and other positive capacities may accumulate over time in response to the unique challenges of community-based practice. While this finding aligns with research indicating that seasoned nurses often develop more refined coping strategies through repeated exposure to stressors [56–58], it also raises questions about how best to accelerate the development of PsyCap among newer nurses. Mentorship programs, reflective practice sessions, and structured orientation curricula could potentially bridge this gap by providing targeted support and role modeling for novice community nurses [59, 60].

In comparing these outcomes with previous literature, the study extends existing knowledge regarding the relationship between PsyCap and occupational outcomes by focusing specifically on the community nursing context—a domain that has been relatively understudied [61]. While most previous research on PsyCap has centered on hospital-based nurses or corporate employees, these findings highlight the transferability and importance of PsyCap in less centralized, more variable work settings [62–64]. Notably, the positive correlations observed here are generally in line with those reported in other nursing populations, underscoring the broad relevance of self-efficacy, hope, and optimism as key drivers of psychological well-being and satisfaction in healthcare professionals [65].

While numerous studies have explored the impact of Psychological Capital (PsyCap) on hospital-based nurses, research focusing on community healthcare settings remains relatively limited. Unlike hospital nurses, who operate within structured environments with readily available resources and immediate peer support, community nurses often work independently in diverse and sometimes under-resourced settings. This autonomy, coupled with the need to navigate varied socio-cultural contexts, places greater demands on their coping mechanisms and psychological resilience. Studies examining PsyCap in primary care and home healthcare nurses suggest that higher levels of self-efficacy and resilience are essential for managing unpredictable work conditions and patient interactions [4, 64]. Additionally, research on healthcare workers in rural settings has shown that hope and optimism play a critical role in sustaining motivation despite limited infrastructure and logistical challenges [66]. These findings reinforce the need for targeted interventions that cater specifically to community nurses’ unique stressors, rather than relying solely on hospital-centric models of PsyCap development. Future studies should further examine how PsyCap-building strategies can be tailored to enhance stress management and job satisfaction in non-hospital nursing environments, where support systems and resources differ significantly.

### Implications for practice and policy

This study highlights the importance of Psychological Capital (PsyCap) in stress management and job satisfaction among community nurses, with significant implications for nursing practice, education, and management.

In nursing practice, the findings suggest that integrating PsyCap training into stress management programs can enhance nurses’ ability to cope with occupational stress. Training workshops focused on building self-efficacy, optimism, resilience, and hope can equip nurses with the psychological tools necessary to manage workplace challenges effectively. Moreover, structured mentorship programs and peer support networks could reinforce these attributes, promoting overall well-being and job satisfaction.

Regarding nursing education, incorporating psychological resilience training into nursing curricula could prepare future nurses for the demanding nature of healthcare work. Educational interventions, such as role-playing, case-based learning, and cognitive-behavioral strategies, can help students develop positive coping mechanisms before they enter the workforce. Additionally, integrating stress management techniques within academic programs could foster emotional intelligence and adaptability, ensuring that graduates are better equipped to handle workplace stressors.

From a nursing management perspective, hospital leadership should consider implementing PsyCap development programs as part of their staff well-being initiatives. Strategies such as leadership coaching, regular resilience-building workshops, and recognition programs can contribute to a supportive work environment. Additionally, fostering a positive organizational culture that values psychological well-being and professional growth can lead to improved nurse retention, reduced burnout, and enhanced patient care quality.

### Study limitations

While the present findings are compelling, several limitations merit consideration. First, the cross-sectional design does not allow for causal inferences. Longitudinal or experimental studies would be necessary to confirm whether bolstering PsyCap indeed leads to improved stress management and higher job satisfaction over time. Second, data were collected from a single site (Tanta University Educational Hospitals), and findings may not generalize to other geographical regions or healthcare institutions. Future research might replicate this work in varying community healthcare settings to bolster external validity. Third, although standardized, validated Arabic measures were employed, self-report instruments are inherently susceptible to social desirability bias. Participants might have overestimated their positive traits or coping abilities, thereby inflating correlations. Triangulating self-report data with supervisor assessments or objective performance indicators could add depth and reliability to subsequent investigations.

### Directions for future research

Building on these observations, longitudinal research designs could explore how PsyCap and stress management strategies evolve over time in response to professional development programs, shifts in healthcare policy, or changes in community health demands. Intervention studies that compare different training modalities (e.g., cognitive-behavioral skills training vs. peer support groups) would be invaluable in determining which approaches most effectively bolster PsyCap among community nurses. Additionally, given the prominent role of hope and optimism in predicting both stress management and job satisfaction, future research might investigate whether these traits can be selectively enhanced or whether they are more effectively developed as part of a broader PsyCap intervention program. Finally, examining the interplay of organizational climate and individual PsyCap could help clarify how supportive leadership, teamwork, and resource allocation modulate the relationships identified in this study.

## Conclusion

In summary, this research highlights the importance of Psychological Capital—particularly self-efficacy, hope, and optimism—in shaping both stress management and job satisfaction among community nurses. By revealing that PsyCap is associated with these outcomes, the study underscores the multifaceted nature of nurse well-being in community settings. The evidence that PsyCap partially works through coping strategies to affect job satisfaction points to the potential efficacy of interventions targeting both personal and organizational factors. Ultimately, investing in the development of nurses’ positive psychological resources could yield substantial benefits, from improved care quality and patient satisfaction to enhanced nurse retention and career longevity. As community nursing continues to expand and adapt to emerging public health challenges, fostering a resilient and resourceful nursing workforce will remain a top priority.

## Data Availability

The datasets generated during and/or analyzed during the current study are available from the corresponding author on reasonable request.
